# Transcriptional characterisation of the *Exaiptasia pallida* pedal disc

**DOI:** 10.1186/s12864-019-5917-5

**Published:** 2019-07-12

**Authors:** Peter A. Davey, Marcelo Rodrigues, Jessica L. Clarke, Nick Aldred

**Affiliations:** 1 School of Natural and Environmental Sciences, Newcastle University, Newcastle upon Tyne, NE1 7RU UK; 2 Brookes Bell, Liverpool, L2 3YL UK

**Keywords:** Anemone, Adhesion, Exaiptasia, Bioadhesion, Cnidarian, mRNA-Seq, Transcriptomics

## Abstract

**Background:**

Biological adhesion (bioadhesion), enables organisms to attach to surfaces as well as to a range of other targets. Bioadhesion evolved numerous times independently and is ubiquitous throughout the kingdoms of life. To date, investigations have focussed on various taxa of animals, plants and bacteria, but the fundamental processes underlying bioadhesion and the degree of conservation in different biological systems remain poorly understood. This study had two aims: 1) To characterise tissue-specific gene regulation in the pedal disc of the model cnidarian *Exaiptasia pallida*, and 2) to elucidate putative genes involved in pedal disc adhesion.

**Results:**

Five hundred and forty-seven genes were differentially expressed in the pedal disc compared to the rest of the animal. Four hundred and twenty-seven genes were significantly upregulated and 120 genes were significantly downregulated. Forty-one condensed gene ontology terms and 19 protein superfamily classifications were enriched in the pedal disc. Eight condensed gene ontology terms and 11 protein superfamily classifications were depleted. Enriched superfamilies were consistent with classifications identified previously as important for the bioadhesion of unrelated marine invertebrates. A host of genes involved in regulation of extracellular matrix generation and degradation were identified, as well as others related to development and immunity. Ab initio prediction identified 173 upregulated genes that putatively code for extracellularly secreted proteins.

**Conclusion:**

The analytical workflow facilitated identification of genes putatively involved in adhesion, immunity, defence and development of the *E. pallida* pedal disc. When defence, immunity and development-related genes were identified, those remaining corresponded most closely to formation of the extracellular matrix (ECM), implicating ECM in the adhesion of anemones to surfaces. This study therefore provides a valuable high-throughput resource for the bioadhesion community and lays a foundation for further targeted research to elucidate bioadhesion in the Cnidaria.

**Electronic supplementary material:**

The online version of this article (10.1186/s12864-019-5917-5) contains supplementary material, which is available to authorized users.

## Background

Adhesion is ubiquitous in nature, having evolved in all kingdoms ranging in length-scale from macromolecule to whole organisms, and for applications as diverse as cell-cell adhesion, biofilm formation, locomotion, surface attachment, prey-capture, reproduction and the formation of protective casings [9, 31, 35, 86]. The ability of some organisms to attach themselves to surfaces, either permanently or temporarily, has received particular attention. Marine organisms have been highly represented in such studies due to their impressive ability to circumvent environmental conditions that challenge synthetic adhesives [78]. Having evolved independently on numerous occasions [9, 44, 67], bioadhesion has provided organisms from single-cell prokaryotes to complex vertebrates with the adaptability to specialise and survive in niche environments.

Most organisms adhering to surfaces do so via a complex interplay of morphology, behaviour and chemistry. A liquid secretion is often involved, the composition of which can be highly variable and may or may not harden post-secretion. Adhesion to surfaces has been studied, to varying degrees, in sponges [41], freshwater hydrozoans (e.g. *Hydra spp*.; [80, 82]), polychaete worms (e.g. *Phragmatopoma californica*; [48]), gastropod molluscs (e.g. *Arion* spp.; [87]), bivalve molluscs (e.g. *Mytilus spp.*; [85, 99]), barnacles (e.g. *Pollicipes* & *Balanus spp.*; [37, 79, 88]), caddisfly larvae (*Stenopsychie marmorata*; [91]), echinoderms [32, 33], and tunicates [1, 108] to name several. The diversity of metazoan taxa in this restricted list demonstrates not only the importance of bioadhesion for survival in aquatic environments, but also the interest of the research community in identifying convergent themes that run through the adhesives of unrelated species, and which may provide inspiration for future biotechnological applications.

To identify and characterise ancestral mechanisms of adhesion it is necessary to examine adhesion in basal metazoan species. The phylum Cnidaria includes corals, anemones and jellyfish, among others, and is a sister group of the bilateria that diverged > 500 million years ago [90]. Unlike the Porifera (sponges), cnidarians possess body-axis symmetry, a nervous system [51] and other characteristics of complex eukaryotes. To date, studies of cnidarian species have increased our understanding of animal development [27], neural networks [101], immunity [8] and symbiosis [47]. Although the field of bioadhesion is in its infancy, much can therefore be learned from established cnidarian models (e.g. *Exaiptasia pallida*.; *Hydra magnipapillita*) that have toolkits of molecular techniques available for them.

A recent study of the freshwater hydrozoan *H. magnipapillita* [80] used a combination of transcriptomics, proteomics and in-situ hybridisation to provide foundational knowledge of the molecular regulation of bioadhesion in that species. However, general understanding of cnidarian bioadhesion is lacking and the evolutionary distance between marine cnidarians, such as anemones, and freshwater hydrozoans invites more detailed investigation of marine species. Surprisingly, literature on the adhesion of anemones to surfaces is almost completely absent. Early observations of the swimming anemone, *Stomphia coccinea*, suggested that nematocysts were involved in surface adhesion, whilst locomotion and detachment were controlled by muscle contraction [29]. In *Actinia equina*, it was later suggested that bioadhesion could rely on protein-protein interactions rather than mucopolysaccharides or nematocysts [107]. Only one, small-scale, study exists on the screening and identification of adhesion proteins in the cadherin-catenin complex (CCC) associated with cell adhesion in *Nematostella vectensis* [21]. There is, however, no evidence that this moiety relates directly to surface adhesion and *N. vectensis* is, in any case, a sediment-dweller that does not adhere to solid substrates.

**Fig. 1:**
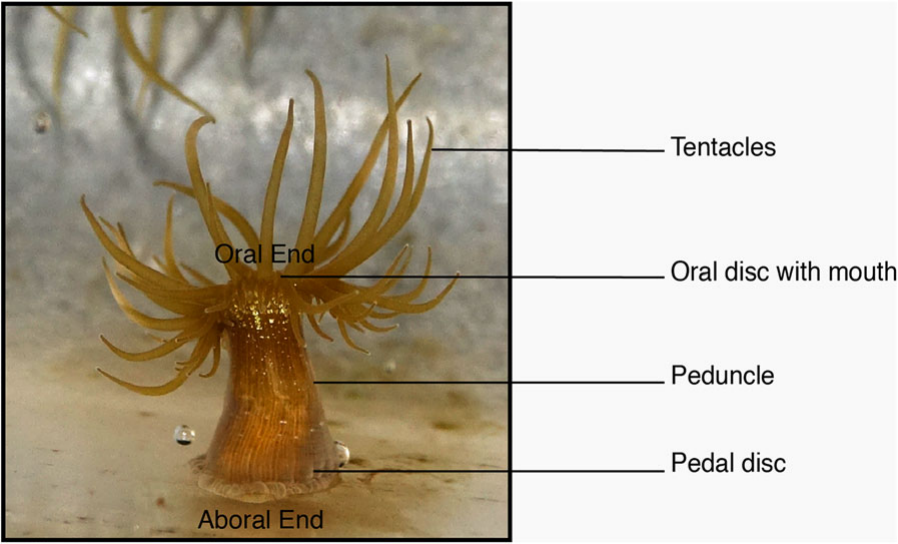
The anatomical body plan of *Exiptasia pallida*. The pedal disc is located at the aboral end of the oral-aboral axis. Image taken by authors of this study

The glass anemone, *Exaiptasia pallida* [38], is a fast-growing, symbiotic species native to shallow waters of the western Atlantic, Caribbean Sea and Gulf of Mexico. Growing to approximately three centimetres when extended, the body plan consists of a pedal disc attached to the substratum, a slender peduncle and an oral disc (with mouth) surrounded by up to 96 tentacles (Fig. 1). Two rows of slits on the peduncle contain nematocyst-armed acontia that extend for defence. Interest in this anemone has grown in recent years for three reasons: 1) Its taxonomic position within the Anthozoa places it close to hard corals and, like many corals, it exhibits a symbiotic relationship with photosynthetic zooxanthellae. It has thus been developed into a model for studying coral-reef, climate-change dynamics; 2) *E. pallida* is easy to culture in the laboratory, making it ideal for manipulation experiments; 3) Publication of the *E. pallida* (strain CC7) genome [13] has provided a suite of DNA sequences, expression-supported mRNAs and predicted proteins for on-going [47, 55, 68] and future high-throughput-omics research.

mRNA-sequencing (mRNA-seq) is a powerful technology for understanding how organisms function, develop and respond to their environments [23], allowing for the detection of novel genes, metabolic pathways, diseases and bioactive compounds [73, 84, 100]. mRNA-seq has been used previously in the field of bioadhesion (e.g. [42, 54]), allowing for interpretation of the genes, molecular processes and regulatory networks associated with adhesion in a number of species [19, 24, 58, 63, 80]. To our knowledge, however, this approach has not been applied to understanding the transcriptional processes involved in adhesion of the anemone pedal disc to surfaces. In this study, a genome reference-assisted transcriptomics approach was taken to identify the regulatory processes associated with the pedal disc of *E. pallida*. The findings highlight the importance of the basal tissue not only for adhesion to surfaces, but also for development, defence and immunity.

## Results

### Transcriptome assembly and differential expression analysis

**Fig. 2:**
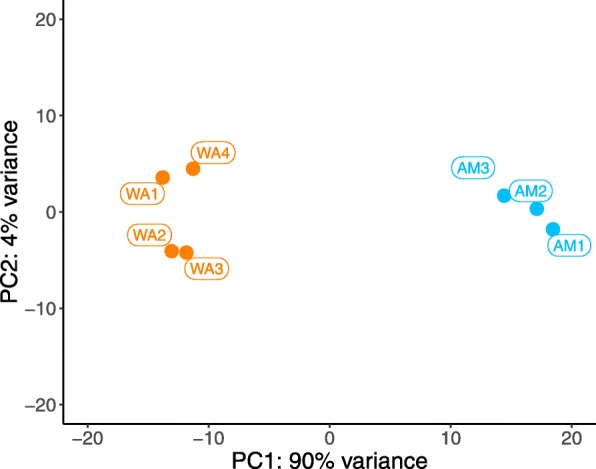
PCA plot showing 94% of the variance between and within groups of mRNA libraries. WA = Whole animal (with pedal disc); AM = Amputated animal (without pedal disc). One of the AM biological replicates was an outlier and as a result it was removed from the experiment (not shown)

After discarding low quality base pairs, rRNA contamination and ambiguous reads, 300,294,692 paired-end reads were uniquely mapped to the *E. pallida* genome [13]. For individual libraries, the percentage of reads that uniquely mapped to the genome ranged from 78.70 to 85.21% (Table 1). Twenty-four thousand, eight hundred and forty-four genes (84.88%) from the genome were found to be expressed in the transcriptome (supported by one or more counts in a library). Of these, 547 (2.20%) genes were differentially expressed, 427 (1.72%) were significantly upregulated and 120 (0.48%) were significantly downregulated in the pedal disc. A principle component analysis (PCA) represents the within and between group similarity of normalised mRNA libraries, with principle components 1 and 2 representing 94% of variability (Fig. 2).

**Table 1:** Number of reads used for the alignment of RNA libraries to the *E. pallida* genome after rRNA removal. Number and percentage of reads uniquely mapped to the *E. pallida* genome. Ambiguous multi-mapped reads were discarded. WA = whole animal, AM = amputated animal (without pedal disc)

RNA Sample	Reads after rRNA removal	Uniquely mapped reads	Uniquely mapped reads (%)
WA_1	67,328,068	57,367,421	85.21%
WA_2	64,708,976	54,799,636	84.69%
WA_3	59,036,216	49,247,662	83.42%
WA_4	51,955,675	43,198,240	83.14%
AM_1	40,391,198	31,786,172	78.70%
AM_2	39,742,533	31,781,245	79.97%
AM_3	40,466,242	32,114,316	79.36%

### Gene ontologies and protein superfamilies of the pedal disc

**Fig. 3:**
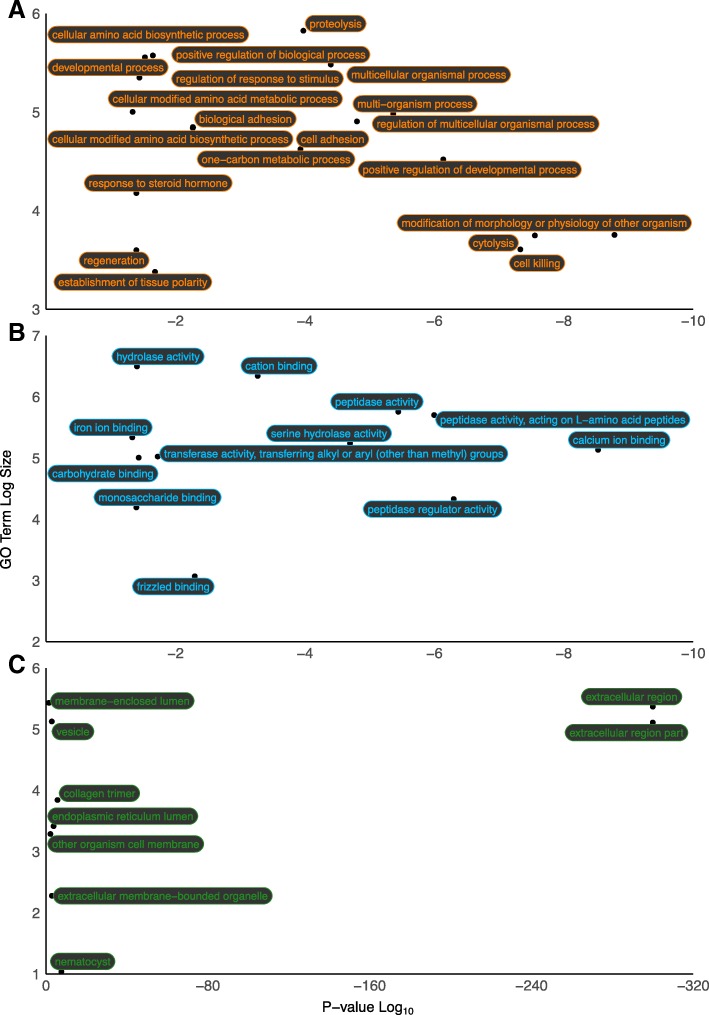
Enriched (FDR: 0.05; ≥ 5 genes per term) gene ontology (GO) terms identified in the pedal disc. Terms are representative of collapsed ReviGO semantic SimRel terms. **a** Biological Processes; **b** Molecular Functions; **c** Cellular Components. Significant enrichment *P*-values (Log_10_) are represented by the X-axis. GO Term Log size (representative gene grouping size) is represented by the Y-axis

A total of 141 GO terms were enriched in the pedal disc (FDR: 0.05; ≥ 5 genes represented by GO term; Additional file 1): 93 biological processes (BP), 24 molecular functions (MF) and 24 cellular components (CC). Collapsing of terms with ReviGO resulted in a total of 41 terms (20 BP; 12 MF; 9 CC; Fig. 3). Of the enriched BP terms (Fig. 3a): modification of morphology or physiology of other organism (GO:0035821), cytolysis (GO:0019835) and cell killing (GO:0001906) were among the most enriched. Further queries of the genes representative of these terms identified genes encoding Matrix metalloproteinase-9, Leukotoxin, toxins (types AvTX, CaTX, CfTX, CrTX, PsTX) and Venom prothrombin activator oscutarin-C non-catalytic subunits. The TX (Toxin) genes identified in this study received a functional annotation to *Actineria villosa* (*AvTX*), *Carybdea alata* (*CaTX*), *Chironex fleckeri* (*CfTX*)*, Carybdea rastonii* (*CrTX*) and *Phyllodiscus semoni* (*PsTX*) homologs. Terms including: regeneration (GO:0031099), positive regulation of developmental process (GO:0051094), developmental process (GO:0032502), response to steroid hormone (GO:0048545), proteolysis (GO:0006508), cellular modified amino acid biosynthetic process (GO:0042398), biological adhesion (GO:0022610) and cell adhesion (GO:0007155) were also enriched.

Among the enriched MF terms (Fig. 3b): Calcium ion binding (GO:0005509), peptidase activity, acting on L-amino acid peptides (GO:0070011), serine hydrolase activity (GO:0017171) and iron ion binding were identified (GO:0005506). Of the nine CC terms identified (Fig. 3c), the terms, extracellular region (GO:0005576) and extracellular region part (GO:0044421) were most enriched. Vesicle (GO:0031982), collagen trimer (GO:0005581), endoplasmic reticulum lumen (GO:0005788), membrane-enclosed lumen (GO:0031974), extracellular membrane-bounded organelle (GO:0065010), other organism cell membrane (GO:0044218) and nematocyst (GO:0042151) were also identified.

Eight condensed GO terms were identified as depleted in the pedal disc (FDR: 0.05; ≥ 5 genes represented by GO term; Additional file 1). These included three BP terms: Protein autoubiquitination (GO:0051865), extracellular matrix organisation (GO:0030198) and extracellular structure organisation (GO:0043062). Two MF terms were depleted: Calcium ion binding (GO:0005509) and extracellular matrix structural constituent (GO:0005201), as well as three CC terms: Extracellular region (GO:0005576), axon (GO:0030424) and extracellular matrix component (GO:0044420). Extracellular matrix structural constituent (GO:0005201) was represented by one Fibrillin-1, two Fibrillin-2, a Mucin-4, a Papillin and four Collagen alpha-1 related genes.

**Fig. 4:**
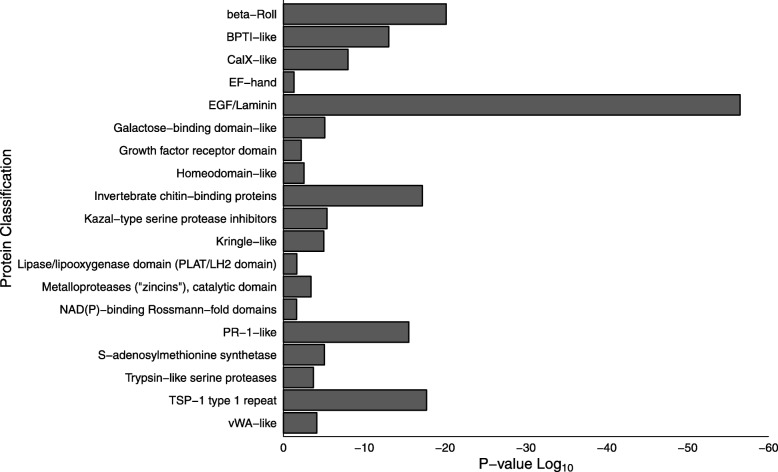
Superfamily protein domain classifications significantly enriched (FDR: 0.05; ≥ 5 genes per classification) in the *E. pallida* pedal disc

Nineteen protein superfamily domain classifications (Fig. 4; Additional file 1) were enriched in the pedal disc (FDR: 0.05; ≥ 5 genes represented by superfamily classification). Top superfamilies included: EGF/Laminin (SCOP: 57196), beta-Roll (SCOP: 51120), Thrombospondin-1 (TSP-1) type 1 repeats (SCOP: 82895), Invertebrate chitin binding proteins (SCOP: 57625) and PR-1-like (SCOP: 55797). Other superfamilies were associated with oxidoreductase, binding and enzymatic functions including Trypsin-like serine proteases (SCOP: 50494; Fig. 4). Eleven superfamilies were depleted in the downregulated gene set (FDR: 0.05; ≥ 5 genes represented by superfamily classification; Additional file 1). Superfamilies depleted in the pedal disc included: Spectrin repeat (SCOP: 46966), Thyroglobulin type-1 domain (SCOP: 57610), Cadherin-like repeats (SCOP: 49313), and Immunoglobulin (SCOP: 48726).

### Upregulated genes of the pedal disc

**Fig. 5:**
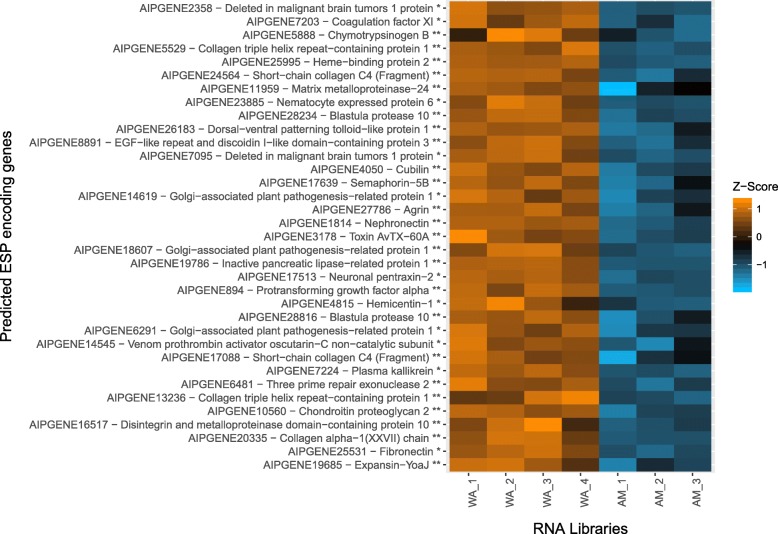
Heatmap depicting the top 35 functionally annotated extracellularly secreted protein (ESP) encoding genes upregulated (Orange) in the pedal disc of *E. pallida.* Z-scores are representative of centred gene expression values (Log_2_). WA_1 – WA_4 = Whole Animal; AM_1 – AM_3 = Amputated animals (pedal disc amputated). * = Non-classically secreted; ** = Classically secreted

There were 427 significantly upregulated genes in the pedal disc, expression differences ranged from 2.00 to 88.65 fold greater (Log_2_ Fold Change (FC) 1.00–6.47). Of those, 250 genes (58.55%) had meaningful functional annotations (e.g not putative, hypothetical, predicted or uncharacterised; [13]). Furthermore, 173 genes (40.52%) were predicted to encode for extracellularly secreted proteins (ESPs). 145 (33.96%) upregulated genes were predicted as containing a signal peptide (classically secreted) and 28 (6.56%) genes were predicted to be secreted without a signal peptide (non-classically secreted). Table 2 and Fig. 5 depict the top 35 genes that received a functional annotation. Eighty-five (49.13%) of the 173 genes had no meaningful functional annotation (as defined above). For a full repertoire of all 173 genes (including those without meaningful functional annotations), see Additional file 1.

**Table 2:** Top 35 ab initio predicted extracellular secreted proteins which received a meaningful functional annotation. Mode = Secretory mode: C = Classical secretory pathway (Predicted by SignalP5.0); NC = Non-classical secretory pathway (Predicted by SecretomeP2.0). FC = Log_2_ fold change of gene expression

Aipgene	Mode	Annotation	FC	Protein superfamily domain (s)
2358	NC	Deleted in malignant brain tumors 1 protein	6.47	1 Spermadhesin, CUB domain,2 SRCR-like
7203	NC	Coagulation factor XI	4.67	1 Trypsin-like serine proteases
5888	C	Chymotrypsinogen B	4.59	1 Trypsin-like serine proteases
5529	C	Collagen triple helix repeat-containing protein 1	4.20	–
25995	C	Heme-binding protein 2	3.82	1 Probable bacterial effector-binding domain
24564	C	Short-chain collagen C4 (Fragment)	3.68	–
11959	C	Matrix metalloproteinase-24	3.51	1 PGBD-like
23885	NC	Nematocyte expressed protein 6	3.36	1 Metalloproteases (“zincins”), catalytic domain
28234	C	Blastula protease 10	3.35	1 Metalloproteases (“zincins”), catalytic domain
26183	C	Dorsal-ventral patterning tolloid-like protein 1	3.26	2 Spermadhesin, CUB domain
8891	C	EGF-like repeat and discoidin I-like domain-containing protein 3	3.14	5 Galactose-binding domain-like
7095	NC	Deleted in malignant brain tumors 1 protein	3.04	–
4050	C	Cubilin	2.75	2 Concanavalin A-like lectins/glucanases,4 Spermadhesin, CUB domain
17639	C	Semaphorin-5B	2.68	3 CalX-like,6 TSP-1 type 1 repeat
14619	NC	Golgi-associated plant pathogenesis-related protein 1	2.53	1 PR-1-like
27786	C	Agrin	2.50	10 Kazal-type serine protease inhibitors
1814	C	Nephronectin	2.47	2 Growth factor receptor domain
3178	C	Toxin AvTX-60A	2.45	–
18607	C	Golgi-associated plant pathogenesis-related protein 1	2.44	1 PR-1-like
19786	C	Inactive pancreatic lipase-related protein 1	2.37	1 alpha/beta-Hydrolases,1 Lipase/lipooxygenase domain (PLAT/LH2 domain)
17513	NC	Neuronal pentraxin-2	2.34	1 Concanavalin A-like lectins/glucanases,1 Integrin alpha N-terminal domain
894	C	Protransforming growth factor alpha	2.33	1 EGF/Laminin
4815	NC	Hemicentin-1	2.33	4 TSP-1 type 1 repeat
28816	C	Blastula protease 10	2.31	1 Concanavalin A-like lectins/glucanases,1 Metalloproteases (“zincins”), catalytic domain,1 TSP-1 type 1 repeat
6291	NC	Golgi-associated plant pathogenesis-related protein 1	2.29	1 PR-1-like
14545	NC	Venom prothrombin activator oscutarin-C non-catalytic subunit	2.25	1 Family A G protein-coupled receptor-like,1 Galactose-binding domain-like
17088	C	Short-chain collagen C4 (Fragment)	2.24	–
7224	NC	Plasma kallikrein	2.23	1 Trypsin-like serine proteases
6481	C	Three prime repair exonuclease 2	2.23	1 Ribonuclease H-like
13236	C	Collagen triple helix repeat-containing protein 1	2.22	–
10560	C	Chondroitin proteoglycan 2	2.17	11 Invertebrate chitin-binding proteins
16517	C	Disintegrin and metalloproteinase domain-containing protein 10	2.13	–
20335	C	Collagen alpha-1(XXVII) chain	2.13	–
25531	NC	Fibronectin	2.13	1 Kringle-like
19685	C	Expansin-YoaJ	2.12	1 Barwin-like endoglucanases,1 Invertebrate chitin-binding proteins,1 PHL pollen allergen

**Fig. 6:**
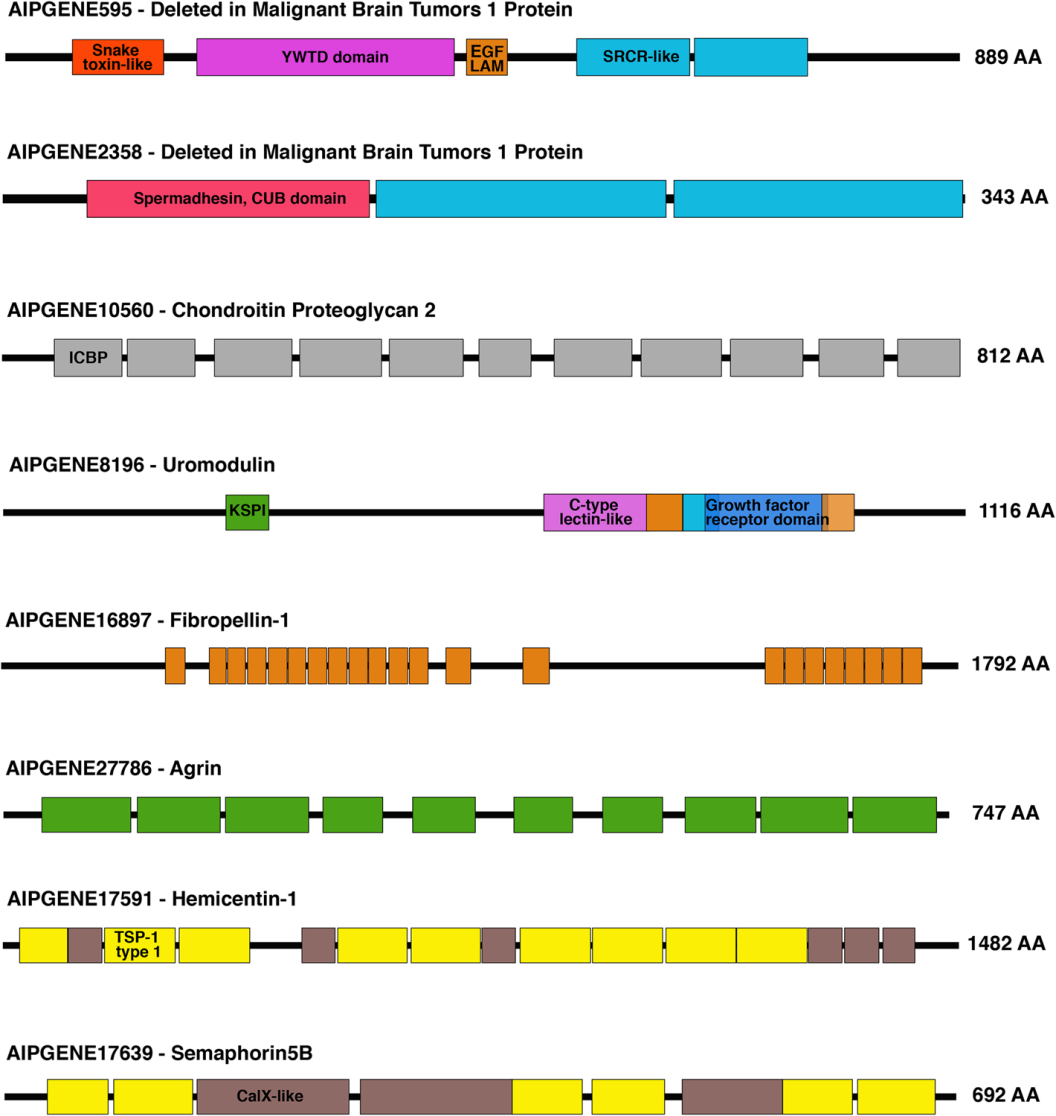
Protein domain architecture of select predicted extracellularly secreted proteins. Length of protein is highlighted as AA (Amino Acids). CUB = Complement C1r/C1s, Uegf, Bmp1; EGF/LAM = Epidermal Growth Factor/ Laminin; SRCR-like = Scavenger receptor cysteine-rich-like; ICBP = Invertebrate chitin binding protein domain; KSPI = Kazal-type Serine Protease Inhibitor; TSP-1 type 1 = Thrombospondin-1 type 1; YWTD = YWTD AA repeat; Colour of domain classifications are consistent with labelling. Aipgene8196 contained overlapping domains as shown

The most significantly upregulated gene in the pedal disc (FC 6.47; Aipgene2358; Deleted in malignant brain tumors 1 protein; *DMBT1*), encoded an ESP containing a single spermadhesion-type CUB domain and two scavenger receptor cysteine-rich (SRCR) domains (Fig. 6). Aipgene7095 (FC 3.04) and Aipgene595 (FC 1.55; Fig. 6) also encoded *DMBT1* homologs.

Fifteen upregulated genes had collagen-related functional annotations. Seven of these encoded for ESPs, including: Aipgene20335 (Collagen alpha-1(XXVII) chain; FC 2.13), Aipgene20425 (Collagen alpha-1(XII) chain; FC 1.77), Aipgene14299 (Collagen alpha-1(XII) chain; FC 1.61), Aipgene17088 (Short-chain collagen C4 (Fragment); FC 2.24), Aipgene24564 (Short-chain collagen C4 (Fragment); FC 3.68); Aipgene5529 and Aipgene13236 (Collagen triple helix repeat-containing protein 1, *CTHRC1*; FC 4.20; FC 2.22). Aipgene 20,425 and Aipgene14299 contained von Willebrand Type A (VWA) domains.

Nine metalloproteinase genes were identified encoding for ESPs. These included: Aipgene11959 (Matrix Metalloproteinase-24; FC 3.51), Aipgene16517 (Disintegrin and metalloproteinase domain-containing protein 10; FC 2.13), Aipgene9574 (Zinc metalloproteinase nas-13; FC 2.07), Aipgene1297 (Matrix metalloproteinase-9; FC 1.62), Aipgene25980 (MAM and LDL-receptor class A domain-containing protein 1; FC 2.01), Aipgene23879 and Aipgene23885 (Nematocyte expressed protein 6 (NEP-6); FC 1.71, 3.36), Aipgene28234 and Aipgene28816 (Blastula Protease 10; FC 3.35, 2.31).

Extracellularly secreted glycoproteins and proteoglycans were found to be encoded by upregulated genes. These included: Aipgene8196 (Uromodulin; FC 1.59; Fig. 6), Aipgene25531 (Fibronectin; FC 2.13), Aipgene23599 and Aipgene23611 (Rhamnose-binding lectin; FC 1.76, 1.37), Aipgene16897 (Fibropellin-1; FC 1.74; Fig. 6), Aipgene14232 (Neurogenic locus notch homolog protein 3; FC 1.93), Aipgene27786 (Agrin; FC 2.50; Fig. 6), Aipgene4050 (Cubilin; FC 2.75) and Aipgene10560 (Chondroitin Proteoglycan 2, FC 2.17; Fig. 6). Aipgene16897 (Fibropellin-1; Fig. 6) contained 22 EGF (Epidermal Growth Factor) repeats, while Aipgene14232 (Neurogenic locus notch protein) contained 7. Although not predicted as being extracellularly secreted in this study, Aipgene16924 (Neurogenic locus notch protein) was differentially expressed and contained 19 EGF repeats. Aipgene10560 (Chondroitin Proteoglycan 2) contained 11 Invertebrate chitin-binding (peritriphin-A) domains. Additionally, five genes encoding extracellularly secreted Semaphorin-5B (one gene; FC 2.68) and Hemicentin-1 (four genes) contained more than one TSP-1 type 1 repeat (Fig. 6; Additional file 1).

In conjunction with the enrichment of GO terms associated with proteases, a host of trypsin-like serine protease genes were identified as encoding ESPs. These included Aipgene7203 (Coagulation factor XI, FC 4.67), Aipgene5888 (Chymotrypsinogen B, FC 4.59), Aipgene7224 (Plasma kallikrein, FC 2.23), Aipgene7188 and Aipgene7180 (Transmembrane protease serine 3; FC 1.91, 1.85), Aipgene7205 (Transmembane protease serine 5; FC 1.88) and Aipgene9301 (chymotrypsin-like protease CTRL-1; FC 1.53). On the contrary, the ESP encoded by Aipgene27786 (Agrin; FC, 2.50) contained 10 kazal-type serine protease inhibitors (KSPI). Aipgene8196 (Uromodulin; FC 1.59) also contained one KSPI.

## Discussion

### Transcriptome assembly

In this study a transcriptome was assembled to enable profiling and characterisation of genes expressed in the pedal disc of *E. pallida*. The high percentage of reads (78.70 to 85.21%) aligning to the genome after quality checks and screening provided ample sequencing depth for a comparative transcriptomic analysis [66]. Analysis of the transcriptional processes up-regulated in the pedal disc provided significant insight into the processes of extracellular matrix (ECM) secretion, adhesion, defence, immunity, and the development of the tissue in contact with the substratum.

### Gene ontology enrichment

The enriched biological process (BP) terms indicated a prominent role for immunity and pathogen defence in the pedal disc. Further queries of the genes associated with toxin production suggested conservation of toxin-encoding sequences throughout species of the Anthozoan and Cubozoan lineages. The majority of toxin genes identified are involved with the formation of the membrane-attack complex/perforin in anemone species [71, 83]. Although prothrombin activators are found in venomous animals, these genes promote thrombin synthesis required for coagulation, cellular aggregation and inflammatory responses in eukaryotes [12]. These genes may play a dual role in defence and cellular aggregation within the pedal disc. Increased defence and immunity have often been associated with adhesive tissues in order to prevent disease and degradation [26, 52]. Until now, most immune studies in the Cnidaria have focussed on *H*. *magnipapillata*, *Aurelia aurita* [18] and corals [74]. These findings are therefore valuable.

Development, regeneration, cellular homeostasis and degradation processes were prominent in the pedal disc. Enriched terms in the pedal disc indicated that cellular turnover rates may be higher than in the rest of the animal, an observation that aligns well with the asexual fission performed at the pedal disc of *E. pallida*. Maintenance of the ECM throughout pedal disc adhesion would also require constant remodelling and protein turnover [81]. This is especially important given that *E. pallida* is somewhat motile, with the ability to reverse its adhesion to a surface facilitating locomotion. The enrichment of terms, biological adhesion (GO:0022610) and cell adhesion (GO:0007155) imply strongly that the ECM is involved in pedal disc adhesion. Furthermore, depletion of ECM-associated terms in the down-regulated genes imply significant changes and ECM remodelling through the regulation of gene expression.

Given that concentrations of calcium in the extracellular space are four to five orders of magnitude greater (typically 1.2 mM) than intercellular concentrations [50], enrichment of calcium ion binding (GO:0005509) is logical, especially when many ECM protein domains, including EGF/laminins, TSP-1, and C-type lectins bind calcium in order to structurally stabilise the ECM [43]. Enrichment of terms associated with peptidase activity, iron ion binding and peptidase regulation in conjunction with enrichment of KSPIs (SCOP: 100895) and Trypsin-like serine proteases (SCOP: 50494) highlight the importance of serine proteases and protease inhibitors in the pedal disc. Indeed, serine-rich proteins are often found within the adhesives of marine invertebrates [30, 32, 37, 99]. Serine-rich proteins confer adhesive and cohesive ability in marine organisms [109]. Furthermore, use of a serine protease resulted in degradation of the cyprid adhesive of *Balanus amphitrite* [2]. Hypothetically, serine proteases would be required within an ECM based attachment/detachment system to disrupt cross-linking of phosphoserine residues [42] and, as discussed, have a recognised role in degrading and remodelling the ECM [39].

**Fig. 7:**
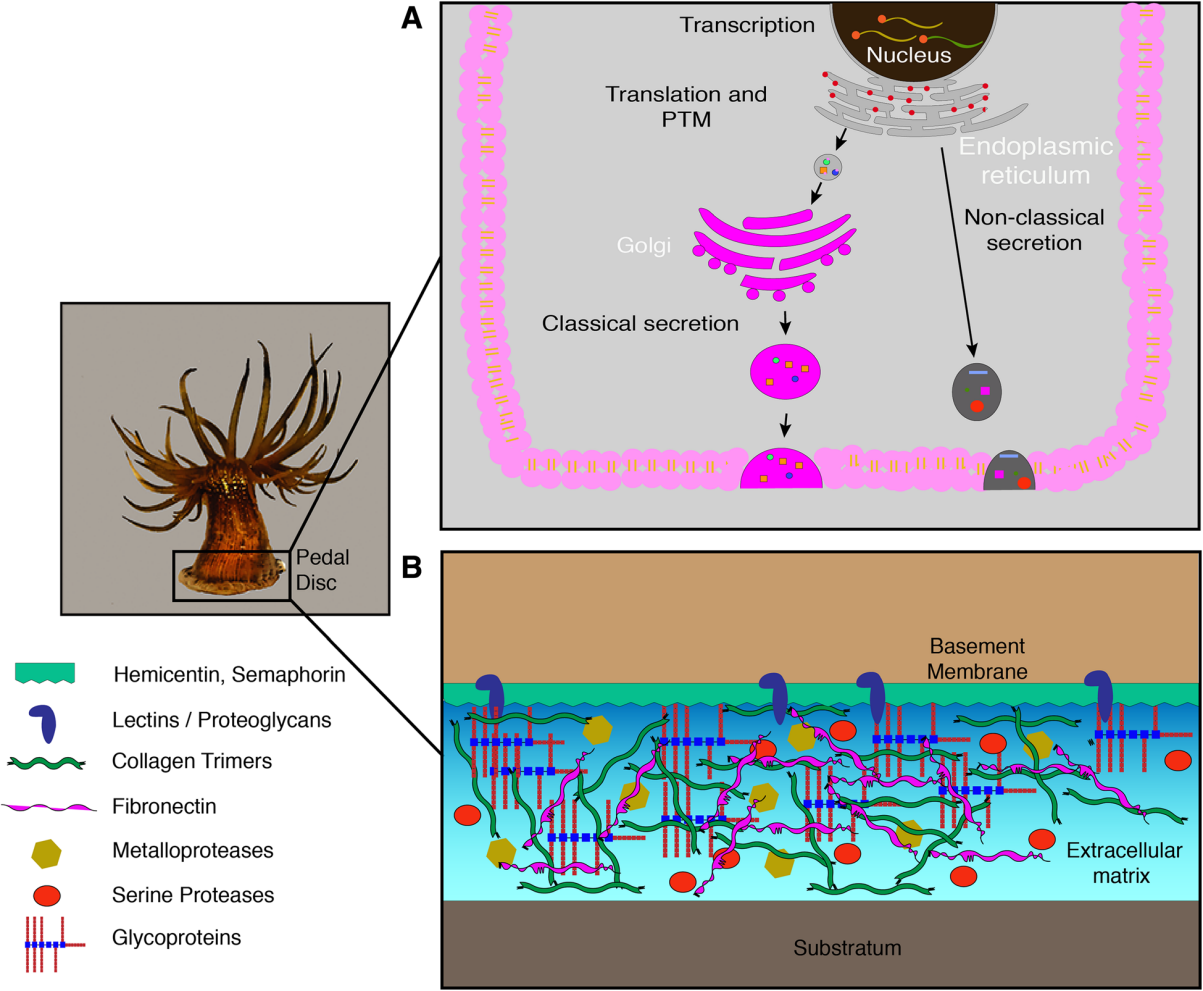
Schematic illustration depicting, **a** Cellular machinery involved in extracellular matrix protein synthesis and trafficking of extracellularly secreted proteins to the extracellular space. **b** ECM protein components suspected to form the basis of pedal disc adhesion in *E. pallida*. Image taken and illustrations created by authors of this study

With respect to enriched CC GO terms, the enrichment of collagen trimer (GO:0005581) is congruent with the literature in which collagen, secreted by fibroblast cells, is one of the major constituents of the ECM [89]. The enriched CC GO terms also imply that the secretory pathway is enhanced in the pedal disc. In theory, for significant extracellular secretion of a proteinaceous matrix, an enriched (larger) endoplasmic reticulum lumen would be required (where post-translational modifications and protein folding occur; [62]), along with increased numbers of vesicles ([97]; Fig. 7). Results of this study are therefore in agreement with those of Young et al.[107] who suggested that adhesion is strongly associated with protein-protein interactions. Overall in comparison to other anemone studies, common GO groups are represented across many different tissue types and not just one type [11, 61, 93]. For example, cell adhesion (GO:0007155) and calcium ion binding (GO:0005509) are represented in the nematosomes, mesentries and tentacles of *Nematostella vectensis* [11]. Statistical enrichment does; however, suggest GO groupings identified in this study are important for the functioning of the pedal disc.

### Protein superfamily enrichment

For metazoans to evolve from single-celled organisms, cell adhesion was obligatory. New protein domains including EGF, TSP-1, C-type lectins, collagen triple-helix domains, laminins, cadherins and integrins evolved to facilitate adhesion [46]. Enrichment of EGF domains in the pedal disc, with high numbers of this domain appearing in extracellularly secreted Aipgene16897 (Fibropellin-1, Fig. 6) and Aipgene14232 (Neurogenic locus notch homolog protein3) proteins is in line with results of other molecular adhesion studies. In the flatworm, *Macrostomum lignano*, the 17 EGF domain-containing protein, Mlig-ap1, exhibits a cohesive function in the adhesive [105]. EGF repeats have also been identified in echinoderm and mussel adhesion [45]. Whilst the large glycoprotein, Fibropellin, forms an ECM layer known as the apical lamina in the sea urchin, *Strongylocentrotus purpuratus* [16] and sea cucumber *Apostichopus japonicus* [10]. Neurogenic locus notch protein is a receptor involved in cell-cell interaction, cell fate and differentiation [49].

Eleven Invertebrate chitin-binding (peritrophin-A) domains were present within Aipgene10560 (Chondroitin proteoglycan 2, FC 2.17, Fig. 6). In *H. magnipapillata*, chitin-binding (peritrophin-A) domains were suggested to play a role in pedal disc adhesion [80]. Proteoglycans are a major constituent of the ECM and basal membrane [75, 94], consisting of a core peptide with heavily glycosylated sidechains. Chitin-binding proteins have been identified in the gastrolith ECM of crayfish where they cross-link to harden the ECM via the oxidation of phenols or catechols [36].

TSP-1 type 1-containing semaphorin glycoproteins are known guidance molecules in axon development, associated with the ECM [4]. Hemicentin-1 (Fig. 6) is a large extracellular glycoprotein of the immunoglobulin family, conserved in the eukaryotic lineage [98]. In *H. magnipapillata*, *N. vectensis* [96], oysters [34] and echinoderm adhesomes, hemicentin homologs have also been identified [102]. This protein supports architectural and structural integrity of animal tissues, including the ECM [106]. Enriched PR-1 (Pathogenesis related 1)-like domains appeared in 13 genes (Golgi-associated plant pathogenesis related protein 1; GAPR1) of this study. The majority of these proteins are located in the Golgi apparatus, however some are known to be secreted extracellularly [70]. Eight were predicted to be extracellularly secreted in this study. These proteins contain cysteine-rich regions associated with innate immunity and allergenic effects. In the Cnidaria they are commonly associated with nematocysts [72]. Closer examination of depleted superfamilies revealed that genes representing some of these classifications were associated with cytoskeletal development.

### Upregulation of specific genes in the pedal disc

Although 58.55% of the upregulated genes in this study had a functional annotation, the remaining proportion had no functional annotation or a meaningful annotation. The lack of annotation calls for further functional characterisation of the *E. pallida* genome. Generating longer reads via a sequencing platform such as PacBio or Oxford Nanopore and improvement of annotation databases has potential to improve the completeness and accuracy of the genome assembly [13]. Like all reference genomes, the *E. pallida* genome assembly and databases will most likely continue to evolve over time [40].

The most differentially expressed gene in the pedal disc, Aipgene2358 (*DMBT1*), contained one spermadhesion type CUB domain and two SRCR protein domains (Fig. 6). Another *DMBT1* homolog identified in this study, Aipgene595, contained additional domains (Fig. 6). The spermadhesion, CUB domain possesses the ability to bind to ligands e.g. carbohydrates, glycosaminoglycans, phospholipids and protease inhibitors [95]. CUB domains are typically found in proteins involved in developmental processes [17]. SRCR domains, consisting of serine-threonine-rich amino acid motifs, are conserved across the metazoan lineage. *DMBT1* has been found to encode for three types of glycoprotein: Deleted in malignant brain tumours 1 protein, salivary agglutinin (DMBT^SAG^) and lung glycoprotein-340 (DMBT1^GP340^; [57]). This protein is a versatile mucin-like molecule that has involvement in epithelial differentiation, agglutination and defence [64]. Given the absence of organs in Cnidaria, it is likely that these glycoproteins serve a role in innate immunity and agglutination of other proteins in the ECM of the pedal disc. *DMBT1* was found to be upregulated in the corals *Acropora millepora* and *Orbicella faveolata* in response to bacterial challenge [104].

Seven genes were predicted to code for extracellularly secreted proteins associated with collagen metabolism: Aipgene20335 (Collagen alpha-1(XXVII) chain), Aipgene20425 and Aipgene14299 (Collagen alpha-1(XII) chain), Aipgene17088 and Aipgene24564 (short-chain collagen C4 (Fragment)), Aipgene5529 and Aipgene13236 (*CTHRC1*). Collagen alpha-1 is the main constituent of type 1 collagen. Short-chain collagen C4 (a component of Type IV collagen) is associated with forming sheet-like structures underlying the basal membranes of epithelial and endothelial tissues, surrounding muscle cells, peripheral nerves and adipocytes [7]. The two genes encoding collagen alpha-1(XII) chains contained von Willebrand Type A domains involved in adhesion [32, 87, 103]. In contrast, the glycoprotein Aipgene5529 (CTHRC1) inhibits collagen synthesis in ECMs and could be involved in a regulatory feedback loop controlling collagen deposition in the pedal disc. This protein is also required for epithelial-mesenchymal transition and cellular migration [59].

Matrix metalloproteinases (MMP) are implicated in the regulation of growth factors and their receptors, cytokines, chemokines, adhesion receptors and cell surface proteoglycans in order to alter cellular responses to the environment [89]. MMPs are required for normal developmental processes, regeneration of tissue and degradation of ECM proteins [6]. Blastula Protease 10 is a member of the astacin family of zinc-dependant endopeptidases and in sea urchins this MMP is involved in development, contains a tyrosine switch and is influenced by calcium binding [22]. NEP-6 metalloproteinases are associated with nematocysts and are thought to have a dual-role, acting as a potassium-channel toxin and degrading ECM proteins [61]. In conjunction with the enrichment of nematocyst (GO:0042151), the identification of NEP-6 genes and GAPR1 genes, an atypical gland-like nematocyst may play a role in pedal disc adhesion or defence [65].

Uromodulin (Tamm-Horsefall protein) was classified as an upregulated ESP. This protein undergoes heavy glycosylation and promotes protein-protein interactions, resulting in gelification [77]. It contained two EGF/laminin and a C-lectin domain (dependant on calcium for binding; Fig. 6). In agreement with the current study, uromodulin mRNA was found to be localised in the distal portion of the aboral pore of *N. vectensis*, where it may play a role in defence. If tissue damage occurs, mRNA accumulates at the wound site and is considered to be involved in regeneration [28]. The upregulated ESP, fibronectin, is another high-molecular weight glycoprotein that binds large numbers of cell adhesion receptors including proteoglycans, growth factors, collagen, integrins, and other ECM proteins to strengthen the structure of the ECM [110]. Additionally, active fibronectin fibrillogenesis is a prerequisite for the deposition of collagen type I to the ECM [53]. Cubilin, also an upregulated ESP in this study, is a receptor found on the cell surface binding galectin-3, a lectin that promotes cell-matrix interactions [69]. Two rhamnose-binding lectins were also classified as upregulated ESPs in this study. In *H. magnipapillata*, six copies of rhamnose-binding lectins were found to be potentially associated with adhesion [80]. This number may of course be exaggerated by the use of de novo transcriptomics in that study, where difficulties can be encountered defining unigenes and splice variants. As discussed by Rodrigues et al. [80], glycan cross-binding has the potential to facilitate non-covalent cross-linking, increasing cohesion and adhesion strength [54]. Agrin was also an upregulated ESP in this study (Fig. 6). It is a large heparan sulphate proteoglycan known to bind laminin and integrins in the ECM, and is involved in postsynaptic clustering [102]. A schematic diagram details some protein classes believed, on the basis of these data, to be involved in the ECM and possibly adhesion of *E. pallida* to surfaces (Fig. 7).

## Conclusion

The findings of this study, in conjunction with past observations, suggest that adhesion of the *E. pallida* pedal disc may be facilitated through the secretion of an ECM-like proteinaceous matrix, containing collagen, glycoproteins, proteoglycans and lectins. Conversely, the metalloproteinases and serine proteinases identified here may play roles in immunity, degradation and remodelling of the ECM to facilitate and prime the detachment of *E. pallida* from surfaces*.* Cross-linking via oxidoreductase reactions may also occur to strengthen adhesion*.* The methods used resulted in a list of high-confidence ab initio predicted extracellularly secreted proteins. Functional characterisation of proteins, morphological analyses and localisation of mRNA are now necessary to validate the predicted role of these elements. This study and the datasets supplied thus provide a foundation for future research investigating the regenerative capability, fission, immunity and, in particular, the bioadhesion capability of *E. pallida*.

## Methods

### Culturing and sampling

Symbiotic *Exaiptasia pallida* (Strain CC7) were cultured and maintained in polycarbonate 5 L containers within a temperature (26 °C) and light-controlled (~ 60 μmol m^− 2^ s^− 1^ 12 h light: 12 h dark) incubator at the School of Natural and Environmental Sciences, Newcastle University. Salinity was maintained at 35 parts per thousand (ppt) using artificial seawater (TropicMarin™). Anemones were fed with stage 1 *Artemia* sp. nauplii three times per week. Artificial seawater was replenished after feeding. Anemones were starved 48 h prior to sampling in order to minimise contaminating molecular artefacts. For RNA samples, three anemones were pooled to form one biological replicate. In total, four biological replicates were used in this study per tissue type. Two tissue types were sampled and snap frozen in liquid nitrogen: Whole animal (WA) and amputated animal (AM), the latter consisting of the entire animal without the pedal disc. Pedal discs were surgically removed using a sterile razor. Amputated animals (minus the pedal discs) were used instead of pedal disc tissue. This method was previously used on *H*. *magnipapillata* [80] to minimise tissue damage.

### Library preparation, sequencing, quality checks and assembly

Total RNA was obtained from RNA samples by homogenisation with TRIzol Reagent® and the use of a Direct-zol™ RNA Miniprep Plus kit (per manufacturer’s guidelines; Zymo Research). In-house quality checks were performed at the Leeds University NGS facility to ensure all samples achieved a RIN (RNA Integrity number) value of 7 or above for high-quality sequencing. mRNA was enriched using an Illumina TruSeq kit and sequenced with an Illumina Nextseq 500 sequencer (Paired-end: 76 bp × 2). Quality of reads was visualised using FastQC version 0.11.8 software [5]. Reads were quality trimmed and any adaptor sequences were removed with BBDuk of the BBTools software package [20]. The following parameters were used in conjunction with the BBDuk adapters.fa reference file: ordered = t; ktrim = r; k = 23; mink = 11; hdist = 1; qtrim = rl; trimq = 10; minlength = 35; tpe; tbo. Although mRNA enrichment was completed prior to sequencing (Illumina TruSeq Kit), a second rRNA screening and removal step was conducted using BBDuk (parameters: k = 31; hdist = 1) in conjunction with the associated custom ribokmers.fa file [20]. The decontaminated reads were aligned to the *E. pallida* genome (Version 1.0; [13]; http://aiptasia.reefgenomics.org/download/) using STAR ultrafast aligner software (Version 2.7; [25]). A genome index was created with the settings: sjdbGTFtagExonParentTranscript Parent and sjdbOverhang = 75. The largest intronic region of the genome was calculated in conjunction with the mRNA .fasta and .gff3 files of the version 1.0 genome and, as a result, the option alignIntronMax = 70,000 was used as an additional alignment parameter. Subread Featurecounts software (version 1.6.4; [56]) was used to obtain a summary of gene counts for all samples against the genome. The count matrix was imported into R Studio (Version 3.5.3; “Great Truth”). Reads with no evidential support were discarded (0 counts in all libraries). DESeq2 software (Version 1.22.2; [60]) was used to perform normalisation and differential gene expression analysis. Genes were identified as differentially expressed according to the following criteria: *P*-value = 0.05; alpha = 0.05; LogThreshold = 1(log_2_ based value). Additional file 2 depicts the bioinformatics workflow utilised in this study. Raw sequencing reads were deposited under the NCBI Sequence Read Archive (SRA) accession number: PRJNA540572.

### Gene ontology and protein superfamily enrichment analysis

The sub-sets of significantly upregulated and downregulated genes were defined as the foreground datasets and the entire expressed transcriptome of this study was defined as the background dataset for enrichment analyses. Gene ontology and superfamily protein domain classifications were obtained from the Reef Genomics repository and the authors of the genome publication [13]. Fisher’s exact statistical test was conducted within R using the fisher.test function with the option, alternative = “greater”. Multiple comparison correction was performed with an FDR threshold of 0.05 [15]. Additionally GO terms or protein superfamily domain classifications not represented by at least 5 or more differentially expressed genes were discarded to improve confidence. Those enriched GO terms that met the criterion were condensed and visualised using the Revigo GO visualisation web portal (http://revigo.irb.hr/revigo.jsp; [92]). A ‘small’ (0.5) threshold was chosen for SimRel semantic similarity measure, all other parameters were kept as default. Resulting .csv tables were exported and used for graphically plotting the condensed GO terms.

### Identification of genes encoding for putative extracellularly secreted proteins

Classically secreted proteins (CSPs) contain a signal peptide domain located in the N-terminus of the protein, whereas non-classically secreted proteins (NCSPs) do not contain a signal peptide. Rather, they are transported to their end-destination by carrier proteins and lipids [14]. In order to form a list of high-confidence ab initio extracellularly secreted proteins encoded by the significantly upregulated genes, the predicted protein sequences [13] of the upregulated gene set were first scanned with SignalP5.0 [76] with ‘Eukarya’ selected as organism group. Protein sequences which did not possess a signal peptide according to SignalP5.0 were scanned using SecretomeP 2.0 for NCSPs with an applied neural network (NN) threshold of 0.6. Resulting data of identified CSPs and NCSPs was concatenated and scanned using DeepLoc-1.0 which uses deep-learning and neural networks to classify the final location of proteins [3]. ‘Profiles matrix’ was chosen instead of a Blossum62 matrix for searches to increase accuracy. Proteins classified as ‘extracellular’ by DeepLoc-1.0 provided a final set of genes which putatively encoded for extracellular secreted proteins.

## Additional files

Additional file 1: Bioinformatic analyses additional data. (XLSX 195 kb)

Additional file 2: Schematic diagram detailing the bioinformatics workflow used in this study. (DOCX 44 kb)

## Data Availability

Availability and sources of data used in this study are detailed throughout the article.

## Abbreviations

AA: Amino acids
AM: Amputated animal
AvTX: *Actineria villosa* toxin
BP: Biological processes
CaTX: *Carybdea alata* toxin
CC: Cellular components
CCC: Cadherin-catenin complex
CfTX: *Chironex fleckeri* toxin
CrTx: *Carybdea rastonii* toxin
CSPs: Classically secreted proteins
CTHRC1: Collagen triple helix repeat-containing protein 1
CUB: Complement C1r/C1s, Uegf, Bmp1
DMBT1: Deleted in malignant brain tumors 1 protein
ECM: Extracellular matrix
EGF: Epidermal growth factor
ESPs: Extracellularly secreted proteins
FC: Fold Change
FDR: False discovery rate
GAPR1: Golgi-associated plant pathogenesis related protein 1
GO: Gene ontology
KSPI: Kazal-type serine protease inhibitor(s)
MF: Molecular functions
MMP: Matrix metalloproteinases
mRNA: messenger RNA
mRNA-seq: mRNA-sequencing
NCSPs: Non-classically secreted proteins
NEP-6: Nematocyte expressed protein 6
NN: Neural network
PCA: Principle component analysis
ppt: Parts per thousand
PR-1: Pathogenesis related 1
PsTX: *Phyllodiscus semoni* toxin
RIN: RNA integrity number
rRNA: ribsomonal RNA
SCOP: Structural classification of proteins
SRA: Sequence Read Archive
SRCR: Scavenger receptor cysteine-rich
TSP-1: Thrombospondin-1
TX: Toxin
VWA: von Willebrand type A
WA: Whole animal
