# Supplementary material for: Transcriptional characterisation of the Exaiptasia pallida pedal disc
Source: BMC Genomics. 2019 Jul 12;20:581. doi: 10.1186/s12864-019-5917-5 (PMC6626399; doi:10.1186/s12864-019-5917-5)
Supplement: Supplementary file 2 — Schematic diagram detailing the bioinformatics workflow used in this study. (DOCX 44 kb) [file 12864_2019_5917_MOESM2_ESM.docx]

**** Schematic diagram detailing the bioinformatics workflow used in this study.
